# 14q12q13.2 microdeletion syndrome: Clinical characterization of a new patient, review of the literature, and further evidence of a candidate region for CNS anomalies

**DOI:** 10.1002/mgg3.1289

**Published:** 2020-05-16

**Authors:** Emanuela Ponzi, Mattia Gentile, Emanuele Agolini, Emilia Matera, Roberto Palumbi, Antonia Lucia Buonadonna, Antonia Peschechera, Alessandra Gabellone, Maria Fatima Antonucci, Lucia Margari

**Affiliations:** ^1^ Department of Medical Genetics Di Venere Hospital ASL BARI Bari Italy; ^2^ Genetics and Rare Diseases Research Division Bambino Gesù Children's Hospital Rome Italy; ^3^ Basic Medical Sciences Neuroscience and Sense Organs Department University of the Study of Bari “Aldo Moro” Azienda Ospedaliero‐Universitaria Consorziale Policlinico di Bari Bari Italy

**Keywords:** 14q13 microdeletion syndrome, *BAZ1A*, CNS anomalies, *SNX6*

## Abstract

**Background:**

Chromosome 14q11‐q22 deletion syndrome (OMIM 613457) is a rare contiguous gene syndrome. Two regions of overlap (RO) of the 14q12q21.1 deletion have been identified: a proximal region (RO1), including *FOXG*1(*164874), *NKX2‐1*(*600635), and *PAX9*(*167416) and a distal region (RO2), including *NKX2‐1* and *PAX9*. We report a 6‐year‐old boy with mild dysmorphic facial features, global developmental delay, and hypoplasia of the corpus callosum.

**Methods and Results:**

Array‐CGH analysis revealed a 14q12q13.2 microdeletion. We compared the phenotype of our patient with previously published cases in order to establish a genotype–phenotype correlation.

**Conclusion:**

The study hypothesizes the presence of a new RO, not including the previously reported candidate genes, and attempt to define the associated molecular and psychomotor/neurobehavioral phenotype. This region encompasses the distal breakpoint of RO1 and the proximal breakpoint of RO2, and seems to be associated with intellectual disability (ID), hypotonia, epilepsy, and corpus callosum abnormalities. Although more cases are needed, we speculated on *SNX6*(*606098) and *BAZ1A*(*605680) as potential candidate genes associated with the corpus callosum abnormalities.

## Graphical abstract

We report a 6‐year‐old boy with mild dysmorphic facial features, global developmental delay, and hypoplasia of the corpus callosum and 14q12q13.2 microdeletion. In order to establish a genotype‐phenotype correlation we compared the phenotype of our patient with previously published cases, hypothesize the presence of a new RO and attempt to define the associated molecular and psychomotor/neurobehavioral phenotype.

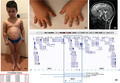

## 1 INTRODUCTION

**FIGURE 1:**
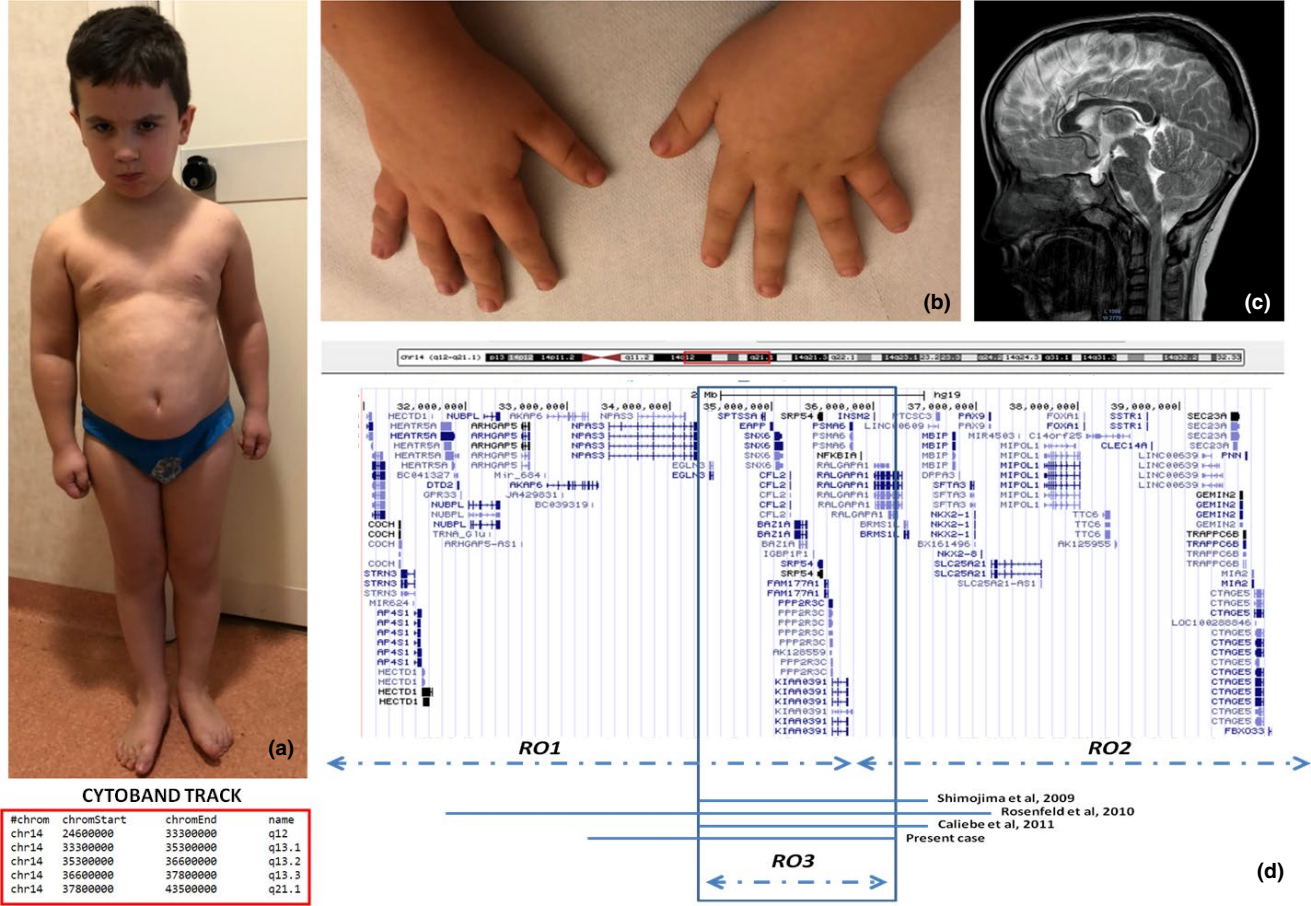
A‒C, Clinical photographs of index case at 6 years of age. Frontal view showing regional abdominal fat distribution (BMI > 99 centile), short neck, and facial features (A); Characteristic hands with stubby fingers (B); Brain magnetic resonance imaging (MRI) sagittal T2 image showing corpus callosum hypoplasia (C). D, Overview of the 14q12q13 deleted region and cytoband track in our case compared with the other patients reported in literature: delineation of the third Region of Overlap (RO3); GeneBank reference sequence for OMIM gene involved in the deleted region for which this data is known: *NPAS3*(NG_013036.2), *CFL2*(NG_012740.1), *PSMA6*(NG_011703.2), *NFKBIA*(NG_007571.1), *RALGAPA1*(NG_051667.1)

Interstitial 14q11‐q22 deletion syndrome (OMIM 613457) is a rare contiguous gene syndrome, with few cases reported in literature (Caliebe et al., 2011; Fonseca et al., 2012; Kamnasaran et al., 2001; Piccione et al., 2012; Santen et al., 2012; Shapira et al., 1994). Although the deletion size is extremely variable, ranging from 3.0 to 40 Mb, and there are no recurrent breakpoints, two regions of overlap (RO), that correlate with disease severity, have been identified (Figure 1D; Santen et al., 2012).

In particular, when the deletion includes the genes *FOXG1*, *NKX2‐1*, and *PAX9*, the prognosis is poor, with severe intellectual disability (ID) and central nervous system (CNS) malformations (i.e., agenesis of the corpus callosum [CC]), while more distal deletions, involving only *NKX2‐1* and *PAX9*, appear to be associated with a milder phenotype (Santen et al., 2012).

Here, we report a de novo 14q12q13.2 microdeletion in a 6‐year‐old boy with global developmental delay, mild dysmorphic facial features, and hypoplasia of the CC. The phenotype of our patient has been compared with other previously reported cases, whose deletions partially or totally overlap the present one, in order to establish a genotype–phenotype correlation in the 14q11‐q22 deletion syndrome and, tentatively, identify a third RO.

## 2 CLINICAL REPORT

The proband, a 6‐year‐old child, was born to Caucasian, nonconsanguineous, phenotypic normal parents. Family history was remarkable for ID and epilepsy in the paternal lineage, and for language delay in the maternal one.

He was born at term by cesarean section, after a normal pregnancy. Birth weight was 4,300 g (>97th centile). No prenatal/perinatal complications were reported. Motor development was slightly delayed, reaching unaided ambulation at 17 months. Language development delay was also observed; he began saying few words at 12 months, but was able to formulate simple sentences only at 3 years of age. At infant school age, the proband had social impairment due to the motor coordination and language difficulties, and deficits in attention.

The patient was referred at age of 5 years. Neurological examination showed generalized hypotonia and severe motor clumsiness. Head circumference was 54 cm (90‒97° centile). He also showed sleep disturbances (difficulty falling asleep and frequent arousals). Verbal communication skills were severely impaired: his expressive language was characterized by phonological deficits and restricted to simple sentences. Verbal reasoning was limited to simple, contextual, and daily tasks. Nonverbal communication was normal. Routine hematologic and biochemical tests, including complete blood count, liver, and renal function tests, were normal. Awake and asleep EEG monitoring showed a normal brain bioelectrical activity.

At the last physical examination, the proband was 6 years and 3 months old, his height was 115 cm (25‒50° centile), his weight 28 kg (>97° centile), body mass index (BMI) 21.2 (99° centile), and the head circumference 55 cm (>97° centile). The parents reported persistent sleep disturbances. Hematologic and biochemical tests (complete blood count, markers of liver and kidney function, serum and urinary amino acids, blood lactate, pyruvate, and ammonium) were normal.

Physical examination showed wide hands with stubby fingers, short neck, high forehead, low setting ears, regional abdominal fat distribution, small lesion along the right mammary line (possible supernumerary nipple), and phimosis (Figure 1A,B). Skeletal examination revealed lumbar hyperlordosis, valgus knees, and general joint laxity. Cardiac examination was normal. Neurological examination displayed generalized hypotonia, diffuse osteotendinous hyperreflexia, bilateral clonus, global motor clumsiness, restlessness, and distal upper and lower limb hyperkinesia.

Brain magnetic resonance imaging (MRI) showed hypoplasia of the CC including the posterior portion (Figure 1C), abnormalities of the lateral ventricles, posteriorly in the cella media, and inferiorly in the trigone, slight enlargement of the subarachnoid space of the frontal convexity. ^1^H‐magnetic resonance spectroscopy (^1^HMRS) of the frontal lobe white matter did not reveal altered metabolites. Neurocognitive assessment included the administration of the Wechsler Intelligence Scale for Children‐Fourth Edition (Wechsler, 2012), which revealed mild intellectual impairment with a total Intelligent Quotient score of 65; the cognitive profile was overall homogeneous, and it was characterized by a slight deficit in the verbal reasoning, with a Verbal Comprehension Index of 62. Scholastic skills showed a global impairment in reading, written expression and mathematics learning. Deficits in adaptive behavior was assessed using the Vineland adaptive behavior scales—Interview Edition (Sparrow, 2003); personal and social autonomy was impaired, with difficulties in social skills and daily activities. A standardized evaluation of the language skills showed impaired verbal expression, limited to simple or incomplete sentences, with several phonological defects (omission or substitution of letters), and poor lexical knowledge. The patient answered to simple and contextual commands; nonverbal communication was normal. Motor coordination ability has been assessed by the administration of the Developmental Test of Visual‐Motor Integration that revealed a mild global impairment of gross and fine motor skills (Beery, Buktenica, & Beery, 2010). The patient behavior was characterized by easy sociability, attention deficits, affective and social immaturity. The profile of the Child Behavior Checklist showed clinical scores in the following areas: “Attention problems,” “Social problems,” and “Thought problems” (Achenbach, 1991).

## 3 GENETIC ANALYSIS

Cytogenetic analysis was performed on QFQ‐banded metaphases (550‐band level): in all the examined metaphases, a normal male karyotype (46, XY) was observed. Genomic DNA was extracted from peripheral blood samples. DNA concentration was measured by fluorimeter (Amersham, Piscataway, NJ) using the Hoechst reagent and adjusted to 400 ng/ml. Array‐CGH analysis was performed using the Cytochip oligo ISCA 4x180K (TechnoGenetics Srl). The analysis revealed an interstitial deletion involving the long arm of chromosome 14: arr[GRCh37] 14q12q13.2(33147358_36088096)x1, size 2.94 Mb (Figure 1D). This result was confirmed by FISH. BAC probes RP11‐379F15 (chr14:34,270,407–34,463,440), RP11‐261B19 (chr14:35,074,841–35,221,993), RP11‐806F7 (chr14:35,740,805–35,923,566; Empire Genomics), mapping in the deleted region, gave only one hybridization signal on the normal chromosome 14. The deletion was not present in the parents. The 2.94‐Mb deleted region contains 16 OMIM genes: *AKAP6*(*6046961), *NPAS3*(*609430; NG_013036.2), *EGLN3*(*606426), *SPTSSA*(*613540), *EAPP*(*609486), *RNU1‐27P*, *RNU1‐28P*, *SNX6*, *CFL2*(*60143; NG_012740.1), *BAZ1A*, *SRP54*(*604857), *PROP*(*601538), *PSMA6*(*602855; NG_011703.2), *NFKBIA*(*164008; NG_007571.1), *INSM2*(*614027), *RALGAPA1*(*608884; NG_051667.1). Therefore, the final karyotype of the patient was designed as (ISCN 2016): 46,XY.arr[GRCh37] 14q12q13.2(33147358_36088096)x1dn.

## 4 DISCUSSION

Here, we report a 6‐year‐old patient with mild ID and hypoplasia of the CC due to a de novo 2.94 Mb microdeletion at the 14q12q13.2 chromosomal region.

Two RO have been identified in the 14q12q21.1 interval: a proximal region (RO1, including *FOXG1*, *NKX2‐1*, and *PAX9*), spanning from 28,000,000 to 35,000,000, associated to severe developmental delay and poor prognosis, and a distal region (RO2, including only *NKX2‐1* and *PAX9*), spanning from 35,000,000 to 40,000,000, associated to a milder phenotype with quite normal psychomotor development (Gentile et al., 2016; Santen et al., 2012; Figure 1D).

In this region, the presence of a holoprosencephaly (HPE) locus (HPE8) had been hypothesized by Kamnasaran, Chen, Devriendt, Mehta, and Cox (2005). However, further evidences established a correlation only with agenesis/or anomalies of the CC and, although CC anomalies might represent a minimal form of HPE, these cannot be considered as part of HPE spectrum (Santen et al., 2012). Therefore, the same authors reevaluated HPE8 as a locus associated with CC anomalies rather than as an HPE locus.

To date, only three patients have been reported with 14q deletion, totally overlapping the interval deleted in our patient and not including *FOXG1*, *NKX1*, and *PAX9* (Figure 1D; Caliebe et al., 2011; Rosenfeld et al., 2010; Shimojima et al., 2009). Shimojima et al. (2009) described one patient with a smaller (2.2 Mb) interstitial 14q13.1q13.3 deletion, and affected by a condition characterized by ID, drug‐resistant seizures, and hypotonia; no dysmorphic features were present. Brain MRI showed mild brain atrophy and electrophysiological examinations, including auditory brain response, visual evoked potential, somatosensory evoked potential, and sensory nerve conduction velocity were all normal. Rosenfeld et al. (2010) described seven individuals; one of them had a deletion overlapping that of our proband. Brain MRI showed hypoplasia of the CC, with no other abnormalities/dysmorphisms. Finally, Caliebe et al. (2011) described a patient with facial dysmorphisms (frontal bossing, pointed chin, deep philtrum), focal epilepsy at age of 4 months, and mildly dilated outer ventricles. The array‐CGH analysis disclosed a 1.9 Mb microdeletion, spanning from 33,740,150 to 35,694,522, not encompassing the *FOXG1*, *NKX1*, and *PAX9*.

Considering these patients and the present case, we hypothesize the presence of a new region of overlap, not including the previously reported candidate genes, associated with a distinct clinical phenotype (Figure 1D). In Table 1 we summarize the clinical phenotype of the cases: CC/CNS abnormalities were present in all cases, developmental delay/ID and hypotonia in 3/4, and 2 patients had seizures. Although the number of patients is still limited, some intriguing observations can be made on the deleted genes potentially candidate in determining the phenotype. *SNX6* encodes for a ubiquitously expressed PX‐BAR protein that plays important roles in retromer‐mediated retrograde vescicular transport from endosomes. SNX6 interacts with Homer1b/c, a postsynaptic scaffold protein crucial for the synaptic distribution of other postsynaptic density proteins and structural integrity of dendritic spines. Recent studies revealed a physiological role of *SNX6* in CNS excitatory neurons. The mild neurodevelopmental phenotype exhibited in mouse model (Nestin‐Cre; Snx6fl/fl mice) with *SNX6FL* haploinsufficiency suggests functional redundancy among the *SNX* family members (Niu et al., 2017). Further studies are needed to characterize the roles of evolutionarily conserved *SNXs*, including *SNX6* and *SNX32*, in sorting and trafficking of neuronal proteins and their functions in the synaptic development and activity. However, this new evidence allows to hypothesize a potential role of this gene in determining the clinical phenotype.

**TABLE 1:** Clinical and genetic synoptic table. Comparison of the clinical and genetic data of the 14q12q13 microdeletion cases reported in literature and our case. NS: not specified

Clinical phenotype	Shimojima et al. (2009)	Rosenfeld et al. (2010)	Caliebe et al. (2011)	Present case
Breakpoint nucleotide position	14q13.1q13.3 33.462.439–35.694.522	14q12q13.3 30.981.266–36.032.919	14q13.1q13.3 33.740.150–35694.522	14q12q13.3 32.217.109–35.157.847
Inheritance	Paternal	NS	De novo	De novo
Sex	F	F	M	M
Neurodevelopmental delay	+	NS	−	+
Microcephaly	−	−	−	−
CC Abnormalities	−	Hypoplasia	NS	Partial hypoplasia
Other CNS abnormalities	Mild brain atrophy	−	−	Lateral ventricles dysmorphisms, slight extension of the subarachnoidal spaces of the frontal convexity
Neurological abnormalities	Hypotonia seizures	−	Hypotonia focal epilepsy	Hypotonia sleep disturbances
Facial dysmorphisms	−	−	Frontal bossing, pointed chin, deep philtrum	Stubby fingers, short neck, high forehead, low setting ears

*BAZ1A* encodes for the chromatin‐remodeling factor ACF1, a member of the ISWI chromatin‐remodeling complexes ACF and CHRAC. ACF1 has been implicated in different functions, including chromatin assembly and remodeling, and in double‐strand DNA damage repair (Ito et al., 1999; Lan et al., 2010). Moreover, ACF1 was recently shown as a critical component in the development of susceptibility to depression, and in regulating stress‐related behaviors (Sun et al., 2015; Zaghlool et al., 2016). Zaghlool et al. (2016) identified, by whole exome sequencing, a missense de novo mutation in *BAZ1A*, in a proband with syndromic ID. Consistent with the function of ACF1 as a chromatin‐remodeling factor, this mutation affects the expression of genes involved in several biological pathways. This data supported the role for *BAZ1A* on the *Wnt* and postsynaptic signaling pathways. Literature database searching by GeneCards (www.genecards.org; Fishilevich et al., 2016) and UniProt (http://www.uniprot.org/—The Uni‐Prot Consortium, 2015) showed that 10 out of these 27 genes are associated to cytoskeleton, integrin and synaptic related pathways, pinpointing the relevance of *BAZ1A* in neural development as supported by the findings of Zaghlool et al. (2016). Recently, Weitensteiner et al. (2018) identified a de novo missense mutation in *BAZ1A*, by whole exome sequencing, in a 11‐year‐old female patient with partial agenesis of the CC, absent septum pellucidum and other malformations resembling the extended spectrum of the VACTER/VACTERL association (Weitensteiner et al., 2018). To further assess the potential role of this gene in human malformations, in situ hybridization studies in mouse embryos between E10.5 and E13.5 were performed. The authors demonstrated that *BAZ1A* is expressed in the cloacal membrane and the central and peripheral nervous system, supposing an involvement of *BAZ1A* in CNS anomalies.

Santen et al. (2012) reported three patients with more distal deletions, only partially overlapping RO3 and not including *SNX6* and *BAZ1A* (Figure 1; Santen et al., 2012). None of these three patients had CNS abnormalities at MRI/US examinations, confirming the plausible pathogenic role of these genes in determining CC abnormalities.

*RALGAPA1* (*GARNL1/TULIP1*), encoding a RaI GTPase activating protein, expressed ubiquitously in pre‐ and postnatal human tissues, particularly in the brain, is a candidate gene for many neurological findings reported in 14q deletion patients (Schwarzbraun et al., 2004). Other several studies have been performed to test the possible pathogenic role of *RALGAPA1*. Shimojima et al. (2009) showed that *RALGAPA1* was highly expressed in zebrafish brain, and knockdown of which resulted in brain developmental delay. However, the pathogenic role of this gene is still debated and more studies will be needed to determine its possible role in the neurologic phenotype of our patient.

Regarding other deleted genes, such as *EAPP*, *CFL2*, *SRP54*, *PPP2R3C*, *PROP*, *PSMA6*, *NFKBIA*, and *INSM2*, to date, there are no reported association with neurodevelopment or anatomical development in humans or animal models.

Our report confirms that 14q12q13.3 deletions are overall rare and variable in size. This interval likely hosts three distinct clinical entities: a proximal deletion (RO1) including the “proximal” region, characterized by severe ID, CNS malformations and poor prognosis, a more distal microdeletion region (RO2), with variable expressivity, associated with mild/absent ID, and a third small overlapping region (RO3), encompassing the distal breakpoint of RO1 and the proximal breakpoint of RO2, expressing with ID, hypotonia, epilepsy, and CC abnormalities (Figure 1D). In this context, we set out to define in detail, for the first time, the molecular and psychomotor/neurobehavioral phenotype associated with this deletion interval. Although more cases are needed, we showed further evidence that *SNX6* and *BAZ1A* represent the potential candidate genes in the pathogenesis of CC abnormalities.

## CONFLICT OF INTEREST

The authors declared that they have no conflict of interest.

## AUTHOR CONTRIBUTION

Emanuela Ponzi contributed to conceptualization/design, methodology, investigation, data curation, formal analysis, resources, and participation in writing and revision. Mattia Gentile contributed to conceptualization/design, methodology, investigation, data curation, formal analysis, resources, supervision/oversight, participation in writing and revision. Emanuele Agolini contributed to supervision/oversight and participation in revision. Emilia Matera, Roberto Palumbi, Antonia Peschechera, Alessandra Gabellone contributed to investigation and clinical data curation and Antonia Lucia Buonadonna Maria Fatima Antonucci contributed to methodology, investigation, data curation, formal analysis. Lucia Margari contributed to the study with supervision/oversight and revision. All authors have approved the manuscript and its submission.

## DECLARATION OF PATIENT CONSENT

The authors certify that they have obtained all appropriate patients consent forms. In the form, the patients have given their consent for their images and other clinical information to be reported in the journal.

## Data Availability

Anonymized data will be shared at request of qualified investigators.
